# Male Gender Is a Contributing Risk Factor to Predict Sticky Fat in Laparoscopic Partial Nephrectomy

**DOI:** 10.3390/life15121913

**Published:** 2025-12-14

**Authors:** Erkan Olcucuoglu, Samet Senel, Muhammed Emin Polat, Kazim Ceviz, Emre Uzun, Mevlut Berk Ceri, Antonios Koudonas, Caglar Sarioglu, Yusuf Kasap, Esin Olcucuoglu, Sedat Tastemur

**Affiliations:** 1 Department of Urology, Ankara Bilkent City Hospital, Ankara 06100, Türkiye; samet_senel_umt@hotmail.com (S.S.); emnplt25@gmail.com (M.E.P.); kazimmzz@hotmail.com (K.C.); emr.uzun.7@gmail.com (E.U.); berkcnsrd@gmail.com (M.B.C.); caglarsarioglu@gmail.com (C.S.); dryusuf85@hotmail.com (Y.K.); sedattastemur@yahoo.com (S.T.); 2 First Department of Urology, Faculty of Urology, Aristotle University of Thessaloniki, 541 24 Thessaloniki, Greece; c3dw9@windowslive.com; 3 Department of Radiology, Ankara Bilkent City Hospital, Ankara 06100, Türkiye; esinolcucuoglu@gmail.com

**Keywords:** sticky fat, male, gender, partial nephrectomy

## Abstract

Objective: This study aimed to identify predictive factors for adherent perinephric fat (APF), or sticky fat, in patients undergoing laparoscopic partial nephrectomy (LPN), with particular emphasis on evaluating male gender as an independent risk factor beyond the Mayo Adhesive Probability (MAP) Score. Materials and Methods: A retrospective analysis was performed on 197 patients who underwent LPN for localized renal tumors between December 2019 and September 2025. Demographic, clinical, radiological, intraoperative, and postoperative variables were collected. Sticky fat was defined intraoperatively. MAP scores were calculated using posterior perinephric fat thickness and fat stranding. Statistical analyses included Mann–Whitney U and Chi-square tests, as well as univariate and multivariate logistic regression (Backward LR method). ROC analysis was used to determine predictive performance. Results: The mean age was 61.3 ± 13.1 years, and 63.5% of patients were male. Sticky fat was observed in 41.6% of cases. Male gender (71.3% vs. 52.4%, *p* = 0.007), older age (62.9 vs. 58 years, *p* = 0.031), and higher MAP scores (*p* = 0.005) were significantly associated with sticky fat. Multivariate analysis identified male gender (OR = 1.97; 95% CI: 1.07–3.61; *p* = 0.029) and MAP score (OR = 1.27; 95% CI: 1.04–1.56; *p* = 0.021) as independent predictors. Combining MAP score and gender improved predictive accuracy (AUC = 0.645). Conclusions: Male gender independently predicts APF, and combining gender with MAP score enhances preoperative assessment and surgical planning.

## 1. Introduction

Renal cell carcinoma (RCC) comprises one of the commonest urological cancers and represents about 2.4% of overall cancer diagnoses among adults [1]. The incidence of RCC has been increasing in recent decades, mostly due to the occasional finding of a renal tumor during radiological imaging performed to clarify other medical conditions [1]. According to the statistical projections, the above increasing trend will continue in the future, which is attributed in part to the even more intensified application of cross-sectional imaging and to the increased effect of recognized risk factors, such as obesity, hypertension, and environmental pollution [1]. RCC affects 50% more men than women, and its prognosis shows a continuous improvement in the last 20 years due to the evolution of surgical methods and the introduction of next-generation immunotherapy and molecular targeting drugs [1].

Regarding the management of localized RCC, the main approach includes the surgical excision of the affected kidney or its affected part, while a subset of selected patients are managed by ablation methods or active surveillance, depending on the patient’s performance status and the tumor characteristics [2]. Tumor size, anatomical complexity, and the presence/absence of a healthy kidney represent the main criteria in the decision-making process for selecting radical or partial nephrectomy (PN) for a patient [2]. In general, both American and European guidelines prioritize PN for cT1 RCC and, if feasible, in patients with solitary kidney, bilateral tumors, or RCC-related familial syndromes [2,3]. On the other hand, large and complex tumors are mostly excised radically by kidney excision, which also depends on the surgeon’s experience and the availability of technical methods that are applied to overcome the technical challenges of PN [2,3]. Over the past 20 years, the use of partial nephrectomy (PN) has increased substantially, and its advantages include a lower risk of early complications—such as acute kidney injury, thromboembolism, and sepsis—as well as reduced overall costs (Pyrgidis et al.) [4]. Based on the above advantages and the technology evolution, a continuous extension of the indications for PN is attempted by renal surgeons to eradicate renal tumors with maximal oncological safety and functional results.

To evaluate the eligibility of a renal tumor for PN, several nephrometry scores (NSs) were developed to standardize and quantify the complexity of the excision and the subsequent tendency for complications. The most applied NSs are R.E.N.A.L (radius, exophytic/endophytic, nearness, anterior/posterior, location), PADUA (Preoperative Aspects and Dimensions Used for an Anatomical Classification), and C-Index (Centrality Index), which are based on the geometrical characteristics of the tumor in relation to the key anatomical structures of the affected kidney [5]. The sum of the above NS demonstrates a significant association with crucial parameters of the perioperative and the early postoperative period, such as blood loss, warm ischemia time, and renal function reduction [5]. Another important parameter demonstrating a strong correlation with an increased risk of complications is the qualitative nature of the perinephric fat, which often requires partial resection or separation from the target anatomical structure (Sempels et al.) [6]. The above properties can be quantified by the Mayo Adhesive Probability (MAP) score, which may predict severe complications during open or laparoscopic PN more precisely than the standard NS [6].

The MAP score was initially introduced by Davidiuk et al. as a radiological image-based predictor of adherent perinephric fat (APF) in PN [7]. APF, also known in the respective literature as sticky or toxic fat, comprises a term to describe the condition of the strong attachment of perirenal fat to the target renal area with subsequent difficulty in resection and a tendency for increased blood loss [7]. MAP score calculation relies on the perirenal fat quantity and density (presence of stranding), and after its initial application in minimally invasive PN, it was also validated in patient cohorts who underwent open PN [8]. More recently, the MAP score was not only associated with APF but also with the long-term sequelae of the extended resection time on the renal function of patients who underwent laparoscopic PF and had APF [9]. Despite the advantage of robotic PN in terms of speed and precision of surgical maneuvers, even the robotic approach produces long-term functional results, which are analogous to the MAP score and the perirenal fat properties [10]. Of note, not only renal surgery but also adrenal surgical procedures are affected by the presence of adherent retroperitoneal fat in terms of perioperative outcomes [11]. The MAP score has also been shown to retain its predictive value in the characterization of periadrenal fat and related perioperative parameters, including operation time and blood loss (Chen et al.) [12].

Therefore, the present study aimed to evaluate and validate the applicability of the MAP score in a single-center cohort of patients undergoing laparoscopic partial nephrectomy. In addition to assessing its predictive performance for adherent perinephric fat (APF), we sought to investigate whether additional clinical or demographic factors could independently predict APF beyond MAP score-based risk stratification. Identifying such factors may contribute to improved preoperative planning, better anticipation of surgical difficulty, and optimization of perioperative outcomes. This study provides further insight into risk assessment strategies for APF in laparoscopic partial nephrectomy and their clinical relevance in routine practice.

## 2. Materials and Methods

### 2.1. Ethical Consideration

The protocol of our study was approved by the local ethic committee and prepared in accordance with the principles of the Declaration of Helsinki (Ethics committee approval number: TABED 2-25-1526).

### 2.2. Study Design, Patient Selection and Data Procedure

We made a retrospective review of the database of patients who underwent laparoscopic partial nephrectomy for a localized renal tumor at our institution from December 2019 to September 2025. There were 216 patients in this survey. Exclusion criteria comprised the absence of adequate preoperative imaging, incomplete clinical or operative data, non-localized renal tumors, and procedures not performed via a transperitoneal laparoscopic approach. Accordingly, 19 patients with unavailable or insufficient preoperative imaging were excluded, and the remaining patients constituted the final study cohort. Finally, 197 patients were included.

Demographic (age, gender, BMI), radiographic (tumor size, localization, side, exophytic rate, posterior perinephric fat thickness, stranding, MAP score), intraoperative (operation duration, amount of bleeding, warm ischemia time, intraoperative complications), and postoperative (malignancy, surgical margin) data and comorbidities (diabetes mellitus [DM], hypertension [HT]) of all included patients were recorded.

Radiographic data were determined from preoperative contrast-enhanced abdominal CT imaging studies obtained before the operation (up to 90 days). Preoperative contrast-enhanced abdominal CT scans have been acquired using different CT scanner models (128 detectors and 512 detectors CT scanner (General Electric, Milwaukee, WI, USA)) within the same institution, according to routine clinical practice. All radiographic parameters, including tumor characteristics and MAP score components, were retrospectively evaluated jointly by one experienced radiologist and one experienced urologist. The assessments were performed in consensus to ensure consistency and reduce interpretation bias. Tumor size indicated the largest diameter of the tumor.

The MAP score was calculated as previously defined by Davidiuk et al. On the axial section at the level of the renal vein, a linear line is drawn from the renal capsule posteriorly, and the thickness is measured (≤1 cm: 0 points; 1–1.9 cm: 1 point; ≥2 cm: 2 points) for fat thickness measurement. Stranding was classified as absent (0 points), type 1 (2 points), or type 2 (3 points); the differentiation is described with illustrative images. The sum of these two scores constitutes the MAP, ranging from 0 to 5 [7].

Sticky fat was assessed intraoperatively by the two operating surgeons. All procedures were performed via a transperitoneal approach by experienced surgeons. Sticky fat was defined as adherent and fibrotic perinephric fat tissue observed during surgery that complicates dissection due to its tight adherence to the renal capsule, in accordance with definitions used in previous studies [13,14,15,16,17]. Thus, the patients were divided into two groups as the sticky fat group and the non-sticky fat group.

### 2.3. Statistical Analysis

Data coding and statistical analyses were performed on the computer using the SPSS 22 software package program (IBM SPSS Statistics, IBM Corporation, Chicago, IL, USA). The conformity of the variables to the normal distribution was examined using the Shapiro–Wilk tests. Normally distributed variables were expressed as means ± standard deviations, and non-normally distributed variables were expressed as median (minimum–maximum) values. The Mann–Whitney U test of non-categorical parameters between groups was used. Chi-square or Fisher’s exact tests were used for categorical variables. Uni-multivariate logistic regression analyses with the Backward LR method were used to reveal the predictive factors for sticky fat in patients undergoing laparoscopic partial nephrectomy. Predictive abilities of significant factors for sticky fat were analyzed with the receiver operating characteristic (ROC) curve at a 95% confidence interval. Cases with a *p*-value below 0.05 were considered statistically significant.

## 3. Results

The mean age of 197 patients was 61.3 ± 13.1 years, and the median BMI was 27.9 (IQR: 25.2–31) kg/m^2^. One hundred twenty-five (63.5%) of the patients were male. Mean tumor size was 3 (IQR: 2.3–3.8) cm. Eight-two (41.6%) of the patients were in the sticky fat group. The rate of male gender was higher in the sticky fat group (71.3% vs. 52.4%, *p* = 0.007). Mean age and posterior perinephric fat thickness were statistically significantly higher in the sticky fat group (62.9 vs. 58 years, *p* = 0.031; 19.5 vs. 12.3 mm, *p* < 0.001). In addition, the operation duration was higher in the sticky fat group (130 vs. 119 min, *p* < 0.001). Intraoperative complication rate was higher in the sticky fat group but the difference was not statistically significant (7.8% vs. 3.7%, *p* = 0.366). Intraoperative complications and their management are summarized as follows. Cases with bleeding requiring transfusion were successfully stabilized after the necessary intraoperative blood replacement, and no additional postoperative treatment was needed. In a subset of patients, re-resection was performed due to suspected residual tumor; this was primarily attributed to adherent perinephric fat and other factors that limited clear intraoperative visualization of the surgical margin. The areas of concern were re-evaluated and resected accordingly, and postoperative assessments confirmed negative surgical margins in all such cases. Additionally, renal artery injury occurred in a small number of patients, for whom conversion to nephrectomy was required to safely complete the procedure. The demographic, radiographic, clinical, radiological, intraoperative, and postoperative characteristics of the patients are summarized in Table 1.

Uni-multivariate logistic regression analyses with the Backward LR method were used to reveal the predictive factors for sticky fat in patients undergoing laparoscopic partial nephrectomy. On univariate analysis, older age (OR = 1.024; 95% Cl = 1.001–1.047; *p* = 0.039), male gender (OR = 2.254; 95% Cl = 1.246–4.076; *p* = 0.007), and higher MAP score (OR = 1.321; 95% Cl = 1.086–1.608; *p* = 0.005) were found as predictive factors for sticky fat. On multivariate analysis, male gender (OR = 1.966; 95% Cl = 1.07–3.612; *p* = 0.029) and higher MAP score (OR = 1.27; 95% Cl = 1.037–1.555; *p* = 0.021) were independent factors in predicting sticky fat (Table 2).

**Figure 1:**
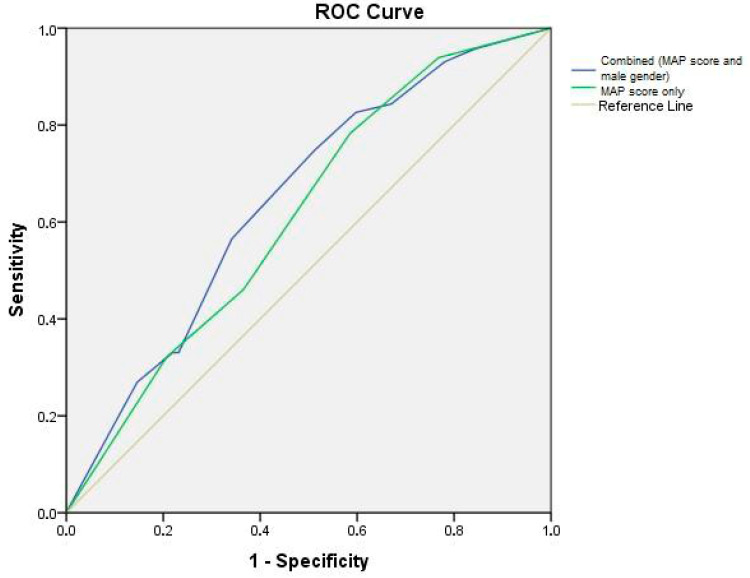
ROC curve analysis.

ROC curves were produced with 95% confidence intervals for demonstrating the predictive value of the MAP score only (AUC = 0.614, CI: 0.533–0.695; *p* = 0.006) and combined parameters (MAP score and male gender) (AUC = 0.645, CI: 0.566–0.724; *p* = 0.001) for sticky fat (Figure 1).

## 4. Discussion

In the current study, we evaluated the retrospective data of a patient cohort who underwent LPN to investigate the effect of the observed APF during the procedure on the perioperative outcomes and to unveil putative predictors of APF presence in a patient. Our results showed that operation duration was significantly prolonged in the APF group, while there were no other significant perioperative differences. APF-positive patients were more frequently men, were older, and had a larger quantity of perirenal fat and higher MAP scores. No differences in metabolic dysfunction-related parameters, such as HT or DM, were found. After performing multivariate analysis, we observed that male gender retained its independent predictive value together with the other important factor, which is the MAP score. This result represents the central finding of our study, since it demonstrates that male gender almost doubles the likelihood of APF regardless of MAP score. Moreover, the above finding suggests that combining the gender data with the MAP score data can further optimize the prediction of APF, as shown in the ROC curve.

From a physiological view, perirenal fat is currently considered not only an anatomical support to the kidney, but also a metabolically active anatomical entity with rich vascularization and autonomic innervation, which plays a role in the balanced function of the renal and cardiovascular system [18]. Perirenal fat is normally more developed in males compared to females, while its further expansion, usually related to obesity, configures an inflammation status, which exerts regional and systemic effects on renal and cardiovascular health [18]. More precisely, inflamed perirenal tissue was recognized as a risk factor of chronic kidney disease, cardiovascular morbidity, and metabolic derangements [18]. In 2025, Chen et al. investigated the presence of metabolic dysfunction in an APF-high risk patient cohort and found that these patients more frequently had HT, DM, and non-alcoholic fatty liver disease in comparison to APF low-risk patients [19]. Interestingly, in our patient cohort, we found no significant differences between APF-positive and APF-negative patients in terms of DM, BMI, and HT.

Regarding the relation of APF to RCC, our literature search showed indications of an ambiguous association. More precisely, APF represents a clinical sign of a metabolic condition, which is accompanied by increased inflammation and HT that are recognized risk factors for RCC, yet there are recent data that correlate pathological perirenal fat (increased quantity/density) with improved survival among RCC patients [20]. Another noteworthy report was published by Kashiwagi et al., who studied the RCC development pattern in relation to perirenal fat characteristics [21]. The researchers found that the increased perirenal fat quantity correlates with increased possibility for outward tumor protrusion, which suggests that perirenal fat may affect the development orientation of RCC [21]. From the above scientific data can be extrapolated that perirenal fat properties seem to play a substantial role not only in terms of tumor excision complexity, but also in RCC emergence, development, and postoperative clinical course.

Regarding the prediction of APF in renal surgery, our literature search demonstrated that in the vast majority of the published reports, the MAP score or single radiological criteria were applied, while a wide differentiation is observed concerning other clinical factors that retain their predictive value independently from the radiological criteria. In 2019, Borregales et al. processed the preoperative and perioperative data of 495 patients who underwent PN and found that DM was an additional factor that could predict APF together with radiological criteria [22]. In another study by Walach et al., APF positivity was defined as an estimated MAP score ≥ 3, and its independent predictive factors were the male gender, BMI ≥ 27.4 kg/m^2^, and an age of ≥65 years [23]. In 2025, Senel et al. evaluated the clinical, radiological, and perioperative data of 238 patients who underwent retroperitoneal LPN, and concluded that APF (as defined intraoperatively by the surgeon) can be predicted by the presence of male gender, DM, or increased perirenal fat density (as estimated radiologically) [24]. From the above-mentioned studies, it can be extrapolated that there is a substantial variation in the research methodology on APF prediction and the additional clinical factors that determine APF risk in combination with perirenal radiological properties.

In our opinion, the results of our study can contribute to an accurate prediction of APF, which may be applied in determining the operative approach and the technical equipment needed for the optimization of oncological and functional results of PN. Intraoperative laparoscopic ultrasound has been shown to be beneficial in patients at high risk for adherent perinephric fat (APF), reducing the duration of perirenal fat dissection, tumor excision, and overall operation time, whereas no significant benefit was observed in APF low-risk patients (Gülşen et al.) [25]. To consolidate the results of our report, further validation in prospective studies is needed.

Potential sources of bias should be considered when interpreting the results of this study, and several limitations should be acknowledged. First, the retrospective design may introduce selection bias, particularly through the exclusion of patients without adequate preoperative imaging. This may have resulted in a study cohort enriched with patients who underwent more standardized preoperative evaluation, potentially leading to an overestimation of the predictive performance of radiologic parameters, including the MAP score. Second, the intraoperative assessment of adherent perinephric fat relied on surgeon judgment, which may introduce observer-related bias. Although this subjectivity could influence the classification of sticky fat, its direction is unlikely to systematically favor a specific patient subgroup. Moreover, given that all procedures were performed by experienced surgeons using a standardized surgical approach, the magnitude of this bias is expected to be limited. Finally, the use of multiple CT scanner models may introduce measurement variability. However, this potential source of bias was mitigated by joint consensus review of all radiologic parameters by an experienced radiologist and urologist, thereby reducing the impact of inter-device variability. Additionally, the absence of standardized quantitative criteria and formal inter-rater reliability analysis for radiologic assessment of fat stranding represents another limitation. Overall, while these methodological limitations and potential sources of bias should be acknowledged, their combined effect is unlikely to substantially alter the main conclusions of the study.

Future studies should focus on prospective, multicenter validation of radiologic prediction models for adherent perinephric fat, incorporating standardized imaging protocols and objective criteria. Integration of advanced imaging analysis and artificial intelligence-based tools may further improve preoperative risk stratification and surgical planning in laparoscopic partial nephrectomy.

## 5. Conclusions

In the current study, we examined the effect of APF on the periprocedural outcomes of PN in a cohort of 197 patients who underwent LPN, and we investigated the putative predictors of APF. Our results showed that APF positivity prolonged the operation duration, but it did not affect the rest perioperative parameters. Among the preoperative factors that were included in the study, radiological properties (expressed as MAP score) were the most robust predictor, while the predictive model was further optimized by the addition of male gender presence, which was the second independent predictive factor of APF.

## Figures and Tables

**Table 1:** Comparison of demographic, radiographic, clinic, intraoperative, and postoperative characteristics according to sticky fat status of patients who underwent laparoscopic partial nephrectomy.

Characteristics	Total (n = 197)	Sticky Fat Group (n = 115, 58.4%)	Non-Sticky Fat Group (n = 82, 41.6%)	*p*
Demographic				
Age (year) (Mean ± SD)	61.3 ± 13.1	62.9 ± 13	59 ± 13.1	**0.031 ^m^**
Gender (male) n, (%)	125 (63.5)	82 (71.3)	43 (52.4)	**0.007 ^c^**
BMI (kg/m^2^) (Median) (IQR)	27.9 (25.2–31)	28.3 (25–31.6)	27.7 (25.2–30)	0.263 ^m^
Radiographic				
Size (cm) (Median) (IQR)	3 (2.3–3.8)	3 (2.2–3.7)	3 (2.4–4)	0.981 ^m^
Localization				
Superior, n (%)	57 (28.9)	32 (27.8)	25 (30.5)	0.896 ^c^
Middle, n (%)	72 (36.5)	42 (36.5)	30 (36.6)	
Inferior, n (%)	68 (34.6)	41 (35.7)	27 (32.9)	
Side				
Left, n (%)	91 (46.2)	54 (47)	37 (45.1)	0.799 ^c^
Right, n (%)	106 (53.8)	61 (53)	45 (54.9)	
Exophytic rate				
≥50%, n (%)	136 (69)	83 (72.2)	53 (64.6)	0.259 ^c^
<50%, n (%)	61 (31)	32 (27.8)	29 (35.4)	
Posterior perinephric fat thickness (mm) (Median) (IQR)	15.8 (7.8–24.5)	19.5 (10.6–27.9)	12.3 (5–19.3)	**<0.001 ^m^**
Presence of stranding				
None, n (%)	94 (47.7)	53 (46.1)	41 (50)	0.44 ^c^
Type 1, n (%)	53 (26.9)	29 (25.2)	24 (29.3)	
Type 2, n (%)	50 (25.4)	33 (28.7)	17 (20.7)	
MAP score (Median) (IQR)	2 (1–4)	2 (2–4)	2 (1–3)	**0.005 ^m^**
Comorbidities				
HT, n (%)	42 (21.3)	27 (23.5)	15 (18.3)	0.381 ^c^
DM, n (%)	30 (15.2)	21 (18.3)	9 (11)	0.191 ^c^
Intraoperative				
Operation duration (min) (Median) (IQR)	125 (105–150)	130 (115–173)	119 (95–140)	**<0.001 ^m^**
Amount of bleeding (mL) (Median) (IQR)	50 (20–150)	50 (20–200)	50 (20–150)	0.233 ^m^
Warm ischemia time, (min) (Median) (IQR)	20 (15–25)	20 (15–25)	19.5 (15–25)	0.544 ^m^
Presence of intraoperative complications, n (%)	12 (6.1)	9 (7.8)	3 (3.7)	0.366 ^f^
Bleeding requiring intraoperative transfusion	3	2	1	
Re-resection requirement for suspected residual tumor	1	1	0	
Renal artery injury	1	0	1	
Conversion to nephrectomy due to failure of accessing to the tumor	5	4	1	
Conversion to open surgery due to failure of dissection of the tumor	2	2	0	
Postoperative				
Malignancy				
Malign, n (%)	142 (72.1)	87 (75.7)	55 (67.1)	0.186 ^c^
Benign, n (%)	55 (27.9)	28 (24.3)	27 (32.9)	
Positive surgical margin, n (%)	3 (1.5)	1 (0.9)	2 (2.4)	0.572 ^f^

BMI: body mass index; HT: hypertension DM: diabetes mellitus; m: Mann–Whitney U test; c: Chi-square test; f: Fisher’s exact test. Bold *p* value characters indicate statistical significance.

**Table 2:** Determination of independent risk factors for sticky fat in patients who underwent laparoscopic partial nephrectomy.

Parameters	Univariate		Multivariate		
	OR (95% CI)	*p*		OR (95% CI)	*p*
Age (per year)	1.024 (1.001–1.047)	**0.039**	Age (per year)	1.018 (0.994–1.041)	0.14
Male gender	2.254 (1.246–4.076)	**0.007**	Male gender	1.966 (1.07–3.612)	**0.029**
BMI (per 1 kg/m^2^)	1.031 (0.973–1.094)	0.3			
Tumor size (per cm)	1.07 (0.864–1.325)	0.535			
Tumor localization (ref. superior)	1				
Middle	1.094 (0.542–2.208)	0.803			
Inferior	1.186 (0.581–2.422)	0.639			
Tumor side (right)	0.929 (0.526–1.64)	0.799			
Exophytic rate (<50%)	0.705 (0.383–1.296)	0.26			
MAP score (per point)	1.321 (1.086–1.608)	**0.005**	MAP score (per point)	1.27 (1.037–1.555)	**0.021**
Presence of HT	1.37 (0.676–2.778)	0.382			
Presence of DM	1.812 (0.783–4.192)	0.165			

CI: confidence interval; BMI: body mass index; MAP: Mayo Adhesive Probability; DM: diabetes mellitus; HT: hypertension.

## Data Availability

The raw data supporting the conclusions of this article will be made available by the authors on request.

## Abbreviations

The following abbreviations are used in this manuscript:

APF	Adherent perinephric fat
BMI	Body mass index
DM	Diabetes mellitus
HT	Hypertension
LPN	Laparoscopic partial nephrectomy
MAP	Mayo adhesive probability
NS	Nephrometry score
PN	Partial nephrectomy
PADUA	Preoperative Aspects and Dimensions Used for an Anatomical Classification
RCC	Renal cell carcinoma
RENAL	radius, exophytic/endophytic, nearness, anterior/posterior, location
ROC	Receiver operating characteristics
